# Clinical features and viral etiology of acute respiratory infection in an outpatient fever clinic during COVID‐19 pandemic in a tertiary hospital in Nanjing, China

**DOI:** 10.1002/jcla.24778

**Published:** 2022-11-29

**Authors:** Yu Geng, Yingying Hao, Xiaoming Xu, Rui Huang, Fei He, Jun Ni, Jie Zhan, Yuxin Chen, FengHua Hu, Chao Wu

**Affiliations:** ^1^ Department of Infectious Diseases, Nanjing Drum Tower Hospital Clinical College of Traditional Chinese and Western Medicine Nanjing University of Chinese Medicine Nanjing China; ^2^ Department of Intensive Care Units Nanjing Drum Tower Hospital, Nanjing University Medical School Nanjing China; ^3^ Department of Emergency Medicine Nanjing Drum Tower Hospital, The Affiliated Hospital of Nanjing University Medical School Nanjing China; ^4^ Department of Laboratory Medicine Nanjing Drum Tower Hospital Clinical College of Jiangsu University, Jiangsu University Nanjing China; ^5^ Department of Infectious Diseases Nanjing Drum Tower Hospital, Clinical College of Nanjing Medical University Nanjing Jiangsu China

**Keywords:** COVID‐19, Viral, acute respiratory infection

## Abstract

**Background:**

Clinical feature and viral etiology for acute respiratory infection (ARI) in the community was unknown during coronavirus disease 2019 (COVID‐19) pandemic.

**Objective:**

In a retrospective study, we aimed to characterize the clinical feature and etiology for the ARI patients admitted to the outpatient fever clinic in Nanjing Drum Tower Hospital between November 2020 and March 2021.

**Methods:**

Fifteen common respiratory pathogens were tested using pharyngeal swabs by multiplex reverse transcriptase‐polymerase chain reaction assays.

**Results:**

Of the 242 patients, 56 (23%) were tested positive for at least one viral agent. The predominant viruses included human rhinovirus (HRV) (5.4%), parainfluenza virus type III (PIV‐III) (5.0%), and human coronavirus‐NL63 (HCoV‐NL63) (3.7%). Cough, sputum, nasal obstruction, and rhinorrhea were the most prevalent symptoms in patients with viral infection. Elderly and the patients with underlying diseases were susceptible to pneumonia accompanied with sputum and chest oppression. Three (5.4%) patients in virus infection group, whereas 31 (16.7%) in non‐viral infection group (*p* = 0.033), were empirically prescribed with antiviral agents. Among 149 patients who received antibiotic therapy, 30 (20.1%) patients were later identified with viral infection.

**Conclusion:**

Our study indicated the importance of accurate diagnosis of ARI, especially during the COVID‐19 pandemic, which might facilitate appropriate clinical treatment.

## Graphical abstract

In a retrospective study, we aimed to characterize the clinical feature and etiology for the ARI patients admitted to the outpatient fever clinic in Nanjing Drum Tower Hospital between November 2020 and March 2021. Fifteen common respiratory pathogens were tested using pharyngeal swabs by multiplex reverse transcriptase‐polymerase chain reaction assays. Of the 242 patients, 56 (23%) were tested positive for at least one viral agent. The predominant viruses included human rhinovirus (HRV) (5.4%), parainfluenza virus type III (PIV‐III) (5.0%), and HCoV‐NL63 (3.7%). Cough, sputum, nasal obstruction, and rhinorrhea were the most prevalent symptoms in patients with viral infection. Elderly and the patients with underlying diseases were susceptible to pneumonia accompanied with sputum and chest oppression. Three (5.4%) patients in virus infection group, whereas 31 (16.7%) in non‐viral infection group (*p* = 0.033), were empirically prescribed with antiviral agents. Among 149 patients who received antibiotic therapy, 30 (20.1%) patients were later identified with viral infection. Our study indicated the importance of accurate diagnosis of ARI, especially during the COVID‐19 pandemic, which might facilitate appropriate clinical treatment.

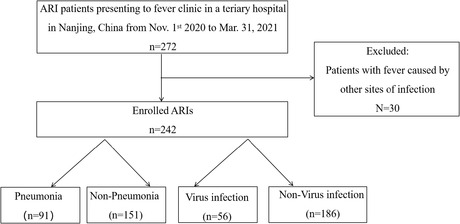

## 1 INTRODUCTION

Acute respiratory infection (ARI) is one of the leading infectious diseases associated with significant morbidity and mortality in the community.^1^ In 2016, global respiratory infections led to 65.9 million hospital admissions, and lower respiratory infections caused 4.4% of deaths in people across all ages.^2^ Influenza‐like illness (ILI) is defined as a sudden onset fever (>38°C) with cough or sore throat in the absence of other diagnoses, accounting for most ARI.^3^ Coronaviruses, respiratory syncytial virus (RSV), influenza A and B viruses, parainfluenza viruses, and adenoviruses are recognized as important viral causes responsible for ARI, which have a high incidence of infection during winter season.^4^, ^5^

The novel severe acute respiratory syndrome coronavirus‐2 (SARS‐CoV‐2) emerged in late 2019 and trigged a worldwide pandemic and caused global health crisis.^6^ The clinical presentation ranges from asymptomatic to mild respiratory tract infection and influenza‐like illness to severe disease with lung injury, multiorgan failure, and death.^7^, ^8^, ^9^ It is well‐established that non‐pharmaceutical interventions and measurements, including wearing masks, hand hygiene disinfection, aggressive detection, quarantine of infected individuals and their close contacts, and social distance, have showed to effectively contain COVID‐19 outbreak. Meanwhile, due to the above non‐pharmaceutical interventions, ARI prevalence during winter season might be partially reduced, and causative agents responsible for ARI might also been changed.^10^, ^11^

ARI are caused by a variety of different pathogens, and there are no effective and specific treatment strategies. Current treatment strategies for ARI include antiviral agent, antitussive, and non‐steroidal anti‐inflammatory drugs, which are administered to relieve the clinical disturbing symptoms. Although antibiotics are not effective against viruses, they are also widely applied as empirical treatment for ARI patients. Herein, our study aimed to identify the viral etiology and clinical features of ARI in adults presenting to fever clinic in a tertiary hospital in Nanjing, China, during COVID‐19 pandemic. Additionally, the clinical characteristics of ARI patients with viral pneumonia versus those without non‐viral pneumonia were also compared.

## 2 METHODS

### 2.1 Study design and respiratory sample collection

This retrospective cohort study included all patients aged over 14 years old who were admitted to the fever clinic of Nanjing Drum Tower Hospital, Nanjing, China, from November 2020 to March 2021 with a clinical diagnosis of ARIs. ARIs defined as patients had a temperature >37.3°C in the past week with one of the following symptoms: sore throat, cough, sputum, runny nose, weakness, or chills. Patients with fever caused by other sites of infection such as acute cholecystitis, central nervous system infection, urinary tract infection, or gastrointestinal tract infection were excluded. The ethics committee approved this study, and written informed consent was obtained from the patients or legal guardians. The respiratory samples and pharyngeal swabs, from patients presenting to the fever clinic, were collected within 1 h of hospital presentation and placed in a 3‐ml tube containing viral preserving solution. The samples were refrigerated at 2–8°C in viral transport medium, transported on ice to the Department of Laboratory Medicine and analyzed immediately or stored at −80°C before analysis. The baseline characteristics, pulmonary computed tomography (CT), clinical manifestations, and clinical diagnoses were retrieved from the electronic medical records of patients.

### 2.2 Respiratory virus Multiplex RT‐PCR assay

Viral nucleic acid from clinical specimens was extracted according to the manufacturer's instruction (DAAN Gene). Briefly, RNA was extracted from 200 μl of each sample with Nucleic Acid Isolation Kit (DA0591), eluted in 50 μl elution buffer, and used as the template for the later assays. Respiratory virus multiplex real‐time reverse transcriptase‐polymerase chain reaction (RT‐PCR) assay detected 15 common respiratory pathogens, including SARS‐CoV‐2, influenza A, influenza B, RSV, adenovirus (AdV), human rhinovirus (HRV), parainfluenza virus type I (PIV‐I), parainfluenza virus type III (PIV‐III), human metapneumovirus (HMPV), human bocavirus (HBoV), enterovirus (EV), human coronavirus (HCoV) NL63/229 E/OC43/HKU1, and Mycoplasma pneumonia, Chlamydia pneumonia, and Legionella pneumophila (Beijing Zhuocheng Huisheng Biotechnology Co).

### 2.3 Statistical analysis

Statistical analysis was conducted using SPSS software version 22.0 (SPSS Inc). Data were presented as means ±SD, median, quartiles, or number (percentage) where appropriate. The chi‐squared test or Fisher's exact was used to compare the difference between groups of dichotomous variables and *t*‐test, ANOVA, or Mann–Whitney *U*‐tests were used in continuous variables. *p* < 0.05 was defined as statistically significant. The underlying pathophysiological status of the patient may affect the blood results for diseases, for example, cirrhosis and hematological disorders. We remove data that may affect the routine blood results and re‐run the statistical analysis to make the results more reliable.

Sensitivity analysis was undertaken in the study. We compared the difference in blood routine test between patients with virus infection and non‐virus infection by excluding participants with underlying diseases such as cirrhosis, leukemia.^12^

## 3 RESULTS

### 3.1 Study Enrollment

**FIGURE 1:**
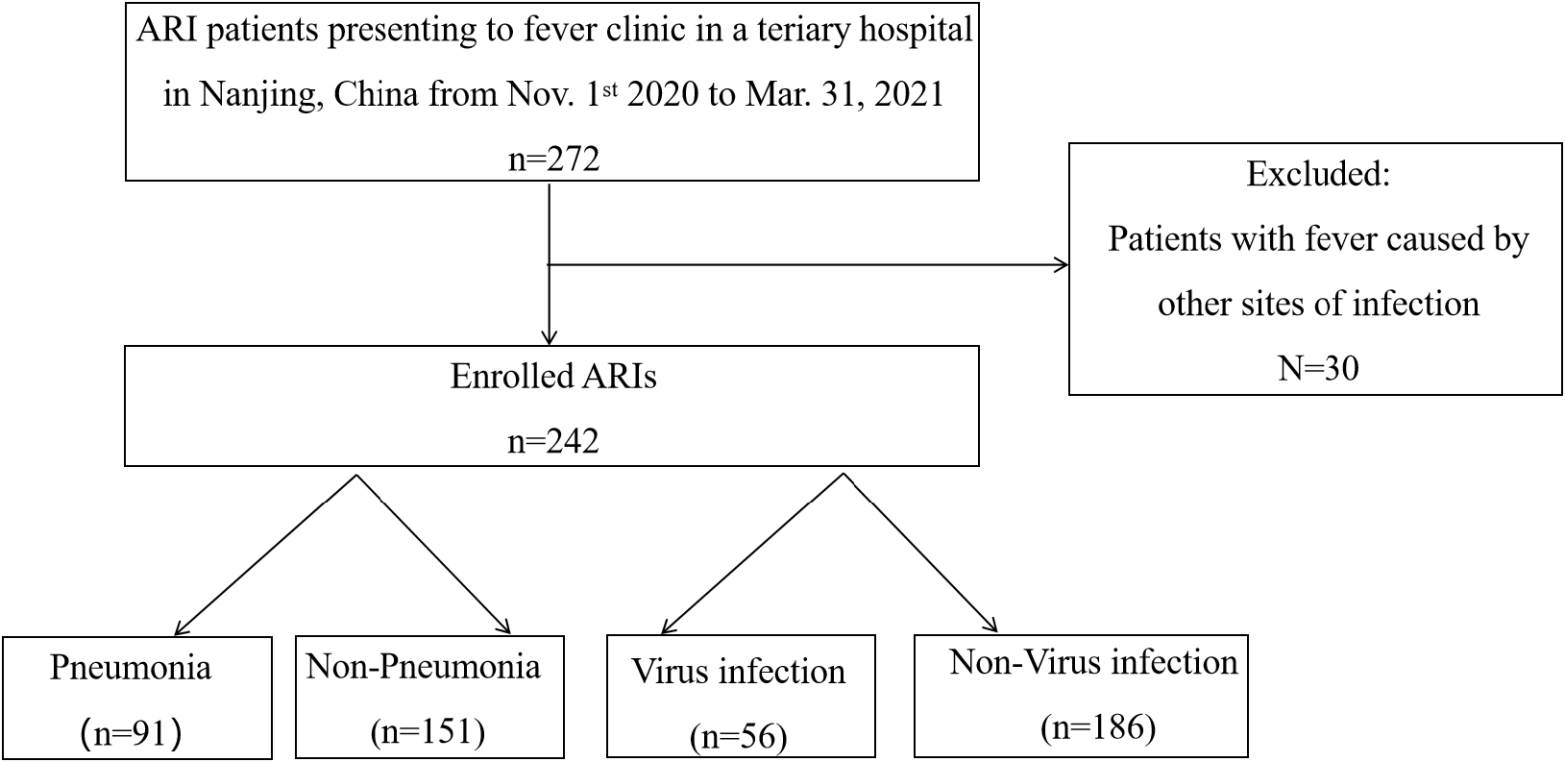
Flowchart of the study.

We consecutively screened 272 outpatients presenting to fever clinic in Nanjing Drum Tower Hospital, Nanjing, China, between November 1, 2020 and March 31, 2021. Among them, 242 cases met the clinical definition of ARIs and, therefore, were included in our retrospective analysis. The remaining 30 outpatients were excluded due to the other sites of the infection. Among 242 enrolled patients, 91 (37.6%) cases were considered as pneumonia due to chest inflammation signs revealed by computed tomography and 73(30.2%) cases were defined as ILIs (Figure 1).

### 3.2 Demographic and clinical characteristics of ARI patients

The demographic and clinical features were summarized in Table 1. The median age of the participants was 30.5 (IQR 23.0–52.2) years and 46.3% (112/242) were male. Fifty‐five (22.7%) patients had a documented or history of underlying diseases, including cardiovascular diseases (12[4.9%]), endocrine system diseases (12[4.9%]), malignant tumor (12[4.9%]), respiratory diseases (7[2.9%]), digestive diseases (9[3.7%]), autoimmune diseases (8[3.3%]), and chronic kidney disease (6[2.5%]). The following clinical symptoms were noted: muscle aches (in 42.5% of patients), weakness (in 40.9%), and sore throat (in 40.1%). Symptoms such as chest tightness (in 4.5%), vomiting (in 5.3%), belly pain (in 2.9%), and diarrhea (in 4.5%) had been much less frequent in the febrile population.

**TABLE 1:** Baseline and clinical characteristics of patients with virus infection and non‐virus infection.

	Virus infection (*n* = 56)	Non‐virus infection (*n* = 186)	Total (*n* = 242)	*p*
Age (years)	34.7 ± 17.5	39.3 ± 20.3	38.2 ± 19.7	0.125
Gender (male)	30 (53.6)	82 (44.1)	112 (46.3)	0.212
Comorbidities	15 (26.8)	40 (21.5)	55 (22.7)	0.408
Respiratory diseases	1 (1.8)	6 (3.2)	7 (2.9)	0.913
Cardiovascular diseases	4 (7.1)	8 (4.3)	12 (5.0)	0.612
Digestive system disease	3 (5.4)	6 (3.2)	9 (3.7)	0.737
Endocrine system diseases	3 (5.4)	9 (4.8)	12 (5.0)	1.000
Neoplastic disorders	4 (7.1)	8 (4.3)	12 (5.0)	0.612
kidney disease	1 (1.8)	5 (2.7)	6 (2.5)	1.000
Immune system disease	3 (5.4)	5 (2.7)	8 (3.3)	0.580
Other diseases	3 (5.4)	5 (2.7)	8 (3.3)	0.580
Fever days	1.2 (0.9, 1.9)	1.2 (0.7, 1.9)	1 (1, 2)	0.937
Clinical features				
Lethargy	13 (23.2)	86 (46.2)	99 (40.9)	0.002
Cough	32 (57.1)	71 (38.2)	103 (42.6)	0.012
Sputum	24 (42.9)	45 (24.2)	69 (28.5)	0.007
Muscle aching	18 (32.1)	85 (45.7)	103 (42.6)	0.072
Pharyngalgia	28 (50)	69 (37.1)	97 (40.7)	0.084
Chills	11 (19.6)	76 (40.9)	87 (36.0)	0.004
Shiver	4 (7.1)	23 (12.4)	27 (11.2)	0.276
Nasal obstruction	24 (42.9)	42 (22.6)	66 (27.3)	0.003
Rhinorrhea	24 (42.9)	43 (23.1)	67 (27.7)	0.004
Headache	19 (33.9)	62 (33.3)	81 (33.5)	0.934
Dizziness	14 (25)	43 (23.1)	57 (23.6)	0.771
Oppression in chest	3 (5.4)	8 (4.3)	11 (4.5)	1.000
Nausea	4 (7.8)	21 (11.3)	25 (10.3)	0.371
Vomiting	3 (5.4)	10 (5.4)	13 (5.4)	1.000
Abdominal pain	2 (3.6)	5 (2.7)	7 (2.9)	1.000
Diarrhea	2 (3.6)	9 (4.8)	11 (4.5)	0.973
Therapy				
Antiviral therapy	3 (5.4)	31 (16.7)	34 (14.0)	0.033
Antibiotic therapy	30 (53.6)	119 (64.0)	149 (61.6)	0.16

We also analyzed the clinical characteristics of patients with pneumonia. The elderly and the patients with underlying diseases were more susceptible to pneumonia (*p* < 0.001). Patients in pneumonia group were more likely to experience sputum (38.5% vs. 22.5%, *p* = 0.008) and oppression in chest (8.8% vs. 2.0%, *p* = 0.032) (Table 2).

**TABLE 2:** Clinical characteristics of patients with pneumonia and non‐pneumonia.

	Pneumonia (*n* = 91)	Non‐Pneumonia (*n* = 151)	Total (*n* = 242)	*p*
Age (years)	49.7 ± 22.2	31.3 ± 14.3	38.2 ± 19.7	<0.001
Gender (male)	46 (50.5)	66 (43.7)	112 (46.3) (46.3)	0.301
Comorbidities	38 (41.8)	17 (11.3)	55 (22.7)	<0.001
Respiratory diseases	6 (6.6)	1 (0.7)	7 (2.9)	0.023
Cardiovascular diseases	10 (11)	2 (1.3)	12 (5.0)	0.002
Digestive system disease	8 (8.8)	1 (0.7)	9 (3.7)	0.004
Endocrine system diseases	9 (9.9)	3 (2)	12 (5.0)	0.015
Neoplastic disorders	6 (6.6)	6 (4)	12 (5.0)	0.546
Kidney disease	4 (4.4)	2 (1.3)	6 (2.5)	0.288
Immune system disease	7 (7.7)	1 (0.7)	8 (3.3)	0.010
Other diseases	2 (2.2)	6 (4)	8 (3.3)	0.706
Virus infection	29 (31.9)	27 (17.9)	56 (23.1)	0.012
Fever days	1.3 (0.8, 2.3)	1.1 (0.9, 1.8)	1 (1, 2)	0.091
Clinical features				
Lethargy	27 (29.7)	72 (47.7)	99 (40.9)	0.006
Cough	45 (49.5)	58 (38.4)	103 (42.6)	0.092
Sputum	35 (38.5)	34 (22.5)	69 (28.5)	0.008
Muscle aching	34 (37.4)	69 (45.7)	103 (42.6)	0.204
Pharyngalgia	28 (30.8)	69 (45.7)	97 (40.7)	0.022
Chills	25 (27.5)	62 (41.1)	87 (36.0)	0.033
Shiver	10 (11)	17 (11.3)	27 (11.2)	0.949
Nasal obstruction	25 (25.3)	43 (28.5)	66 (27.3)	0.154
Rhinorrhea	23 (25.3)	44 (29.1)	67 (27.7)	0.252
Headache	24 (26.4)	57 (37.7)	81 (33.5)	0.069
Dizziness	21 (23.1)	36 (23.8)	57 (23.6)	0.892
Oppression in chest	8 (8.8)	3 (2)	11 (4.5)	0.032
Nausea	9 (9.9)	16 (10.6)	25 (10.3)	0.861
Vomiting	5 (5.5)	8 (5.3)	13 (5.4)	1.000
Abdominal pain	3 (3.3)	4 (2.6)	7 (2.9)	1.000
Diarrhea	3 (3.3)	8 (5.3)	11 (4.5)	0.685

### 3.3 Viral etiology distribution of ARI patients

**FIGURE 2:**
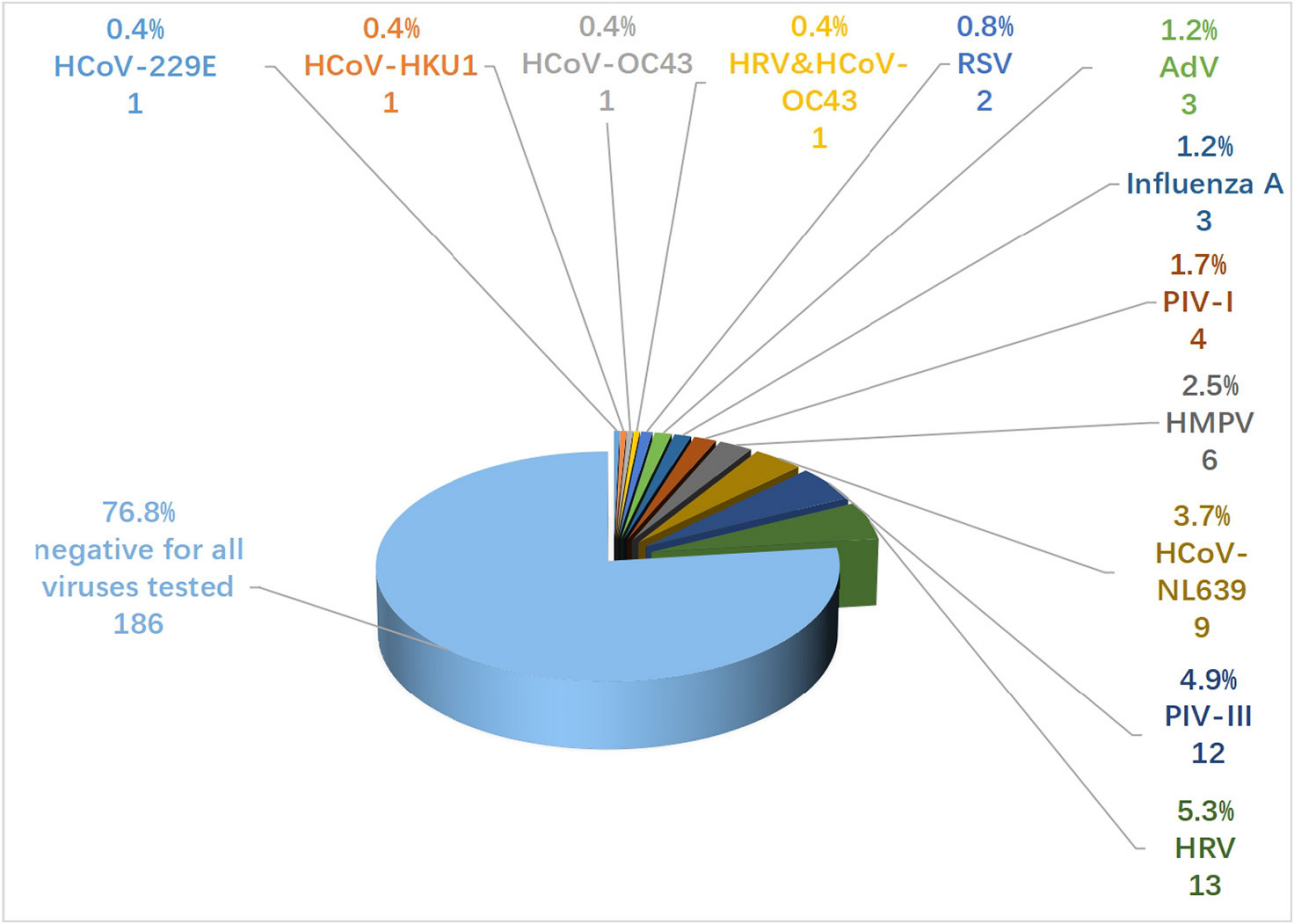
Prevalence of viruses in ARIs patients. Among 242 enrolled patients, 1 (0.04%) case was identified as mixed viral infections of both rhinovirus and coronavirus OC43. 13 (5.4%) patients were HRV viruses, 12 (5.0%) were PIV‐III, and 9 (3.7%) were HCoV‐NL63, 6 (2.5%) were HMPV, 4 (1.7%) were PIV‐I, 3 (1.2%) were Influenza A, 3 (1.2%) were AdV, 2 (0.8%) were RSV, and 1 (0.4%) for HCoV‐OC43, HCoV‐HKU1, and HCoV‐229 E. AdV, adenovirus; HCoV, human coronavirus; HMPV, human metapneumovirus; HRV, human rhinovirus; PIV‐I, parainfluenza virus type I; PIV‐III, parainfluenza virus type III; RSV, respiratory syncytial virus.

All patients were tested negative for SARS‐CoV‐2, and 56 (23%) patients tested positive for other respiratory viruses. 1 (0.04%) case was identified as mixed viral infections of both rhinovirus and coronavirus OC43. Among 55 (22.7%) patients identified with single virus infection, 13 (5.4%) were HRV viruses, 12 (5.0%) were PIV‐III, 9 (3.7%) were HCoV‐NL63, 6 (2.5%) were HMPV, 4 (1.7%) were PIV‐I, 3 (1.2%) were influenza A, 3 (1.2%) were AdV, 2 (0.8%) were RSV, and 1 (0.4%) for HCoV‐OC43, HCoV‐HKU1, and HCoV‐229 E (Figure 2).

ARIs among the elderly could result in advanced severe illness, hospitalization, and even mortality. Therefore, the viral etiology among different age group was further analyzed. The positive rate of respiratory virus was similar among three age groups. Specifically, 29.6% (24/81) of cases were tested positive for viral infection in the 16–25 years old group, 19.6% (19/97) in the 26 to 50 years old group, and 20.3% (13/64) in the group aged over 50 years old, respectively, with no statistically significant differences (*p* > 0.05). The most identified respiratory viruses in the group aged between 14 and 25 years were HRV (8[9.9%]) and HCoV‐NL63 (6 [7.4%]). The major two respiratory viruses detected in the 26–50 years old crew had been PIV‐III (6.2%, 6/97) and HRV (5.2%, 5/97). The most frequent respiratory viruses among patients aged over 50 were PIV‐III (7.8%, 5/64) and HMPV (4.7%, 3/64) (Table 3).

**TABLE 3:** Etiology of acute respiratory infection in different age groups.

Identified virus	14–25 years (*n* = 81)	26–50 years (*n* = 97)	>50 years ( *n* = 64)	Total (*n* = 242)
Rhinovirus	8 (9.9)	5 (5.2)	0 (0)	13 (5.3)
Parainfluenza virus‐I	2 (2.5)	1 (1.0)	1 (1.6)	4 (1.6)
Parainfluenza virus‐III	1 (1.2)	6 (6.2)	5 (7.8)	12 (4.9)
HCoV‐229 E	0 (0)	1 (1.0)	0 (0)	1 (0.4)
HCoV‐HKU1	1 (1.2)	0 (0)	0 (0)	1 (0.4)
HCoV‐NL63	6 (7.4)	1 (1.0)	2 (3.1)	9 (3.7)
HCoV‐OC43	0 (0)	1 (1.0)	0 (0)	1 (0.4)
Respiratory syncytial virus	1 (1.2)	0 (0)	1 (1.6)	2 (0.8)
Influenza A	2 (2.5)	0 (0)	1 (0)	3 (1.2)
Human metapneumovirus	0 (0)	3 (3.1)	3 (4.7)	6 (2.4)
Adenovirus	2 (2.5)	1 (1.0)	0 (0)	3 (1.2)
Rhinovirus and HCoV‐OC43	1 (1.2)	0 (0)	0 (0)	1 (0.4)
Total	24 (29.6)	19 (19.6)	13 (20.3)	56 (23.1)

Additionally, patients with respiratory virus infection had higher percentage of cough (57.1% vs. 38.2%, *p* = 0.012), sputum (42.9% vs. 24.2%, *p* = 0.007), nasal obstruction (42.9% vs. 22.6%, *p* = 0.003), and rhinorrhea (42.9% vs. 23.1%, *p* = 0.004), but lower percentage of lethargy (23.2% vs. 46.2%, *p* = 0.002) and chills (19.6% vs. 40.9%, *p* = 0.004) were less presented, compared with the groups without respiratory virus infection.

### 3.4 Laboratory tests

Blood routine test was performed in all 242 patients. The group identified with viral infection had significantly higher amount of eosinophil compared with non‐virus infection group (0.06 vs. 0.02 × 10^9^/L, *p* = 0.002), while the other blood tests did not show any statistical difference between the two groups. Additionally, ARI patients presenting with pneumonia had significantly lower level of hemoglobin (Hb) compared with ARI patients without pneumonia (133.1 g/L vs. 141.3 g/L, *p* = 0.005) (Table 4 and Table S1).

**TABLE 4:** Blood test analysis of patients with respiratory virus and non‐virus infection groups.

	Virus infection (*n* = 56)	Non‐virus infection (*n* = 186)	*p*
WBC(10^9^/L)	9.65 ± 3.49	10.52 ± 4.25	0.167
Neutrophil (10^9^/L)	7.55 ± 3.32	8.60 ± 4.09	0.083
Lymphocyte (10^9^/L)	1.30 ± 0.66	1.31 ± 0.92	0.965
Monocyte count (10^9^/L)	0.71 ± 0.47	0.66 ± 0.55	0.579
Basophil (10^9^/L)	0.017 ± 0.012	0.015 ± 0.013	0.228
Eosinophil (10^9^/L)	0.06 (0.01, 0.14)	0.02 (0.01, 0.06)	0.002
RBC (10^12^/L)	4.57 ± 0.55	4.61 ± 0.63	0.686
Hemoglobin(Hb) (g/L)	136.96 ± 19.39	138.55 ± 19.17	0.591
Platelet (10^9^/L)	223.60 ± 80.97	217.99 ± 66.07	0.602

Furthermore, sensitivity analysis was conducted since the patients with underlying diseases might affect the blood routine test results and therefore were excluded. Compared with patients with non‐virus infection, neutrophil count was lower (7.49 ± 3.18 vs 8.79 ± 4.07 × 10^9^/L, *p* = 0.037) and eosinophil count was higher (0.06 vs 0.02 × 10^9^/L, *p* = 0.002) in patients with virus infection (Table S2). Meanwhile, there was no statistical difference for blood tests between patients with pneumonia and patients without pneumonia in our sensitive analysis (Table S3).

### 3.5 Therapy and clinical outcomes of ARI patients

Non‐steroidal anti‐inflammatory drugs, corticosteroids, antitussives, and mucolytic agents were used in 149 (61.5%) ARI patients presenting to fever clinic to alleviate fever, soreness of the throat, and cough. Besides, 34 (14.0%) patients were empirically treated with antiviral agents including oseltamivir, peramivir, and ribavirin, including 3(5.4%) patients later diagnosed with viral infection and 31(16.7%) without viral infection (*p* = 0.033). One hundred forty‐nine (61.6%) of 242 patients, including 30 (53.6%) patients identified with viral infection, received antibiotic therapy (Table 1). Of the 242 ARI patients, no patient developed severe pneumonia. Only two (0.8%) patients were admitted to hospital and had recovered fully in a week.

## 4 DISCUSSION

In this study, we investigated clinical characteristics and etiology distribution for patients with ARI presenting to fever clinic in winter season during COVID‐19 pandemic. We found that respiratory virus was responsible for 23.1% of patients with ARI in winter, among which HRV, PIV‐III, and HCoV‐NL63 were the most detected. Our data suggested that the respiratory virus infection rate and etiology were quite different from previous reports, which were previously dominated by influenza A.^13^, ^14^

Firstly, previous epidemiological survey has indicated that most of the respiratory viruses had a seasonal outbreak. Epidemics of influenza virus, RSV, and HCoV peaked in the winter season, while the incidence of enteroviruses peaked in summer.^15^, ^16^, ^17^ Distinct emerging strains of influenza predominated from year to year, while influenza A accounted for more than 50% of flu patients during the influenza season.^18^, ^19^ AdV, HMPV, and HRV can be detected throughout the year, whereas severe cases caused by HRV infection often occurred in winter.^20^, ^21^ Moreover, PIV‐I and PIV‐III were more predominately prevalent in the fall and spring–summer, respectively.^22^

Secondly, the respiratory viral interferences were found at the cellular, host, and population levels.^23^, ^24^ It had been observed that influenza viruses and RSV did not share peaks during the same prevalent period.^23^ Rhinovirus prevalence, for instance, was delayed in the year of 2009 due to the influenza pandemic in Europe.^23^, ^25^ Since COVID‐19 outbreak, a dramatic change in the level of influenza patients was noticed. The rate of influenza infection dropped from 33.8% to 0.6% in southern China, and from 26.5% to 3.2% in northern China, respectively.^26^ The annual infection rate of influenza in southern China has declined by 79.2% and ILI cases were declined by 55%.^27^ In our study, influenza virus was only detected in 3 ARI patients. Such differences before and during COVID‐19 pandemic may be explained by the immunologic mechanism as other respiratory viruses, including disruption of cell surface viral receptor, cell death, the host interferon (IFN) responses, or protective antibody‐driven.^28^, ^29^, ^30^ Thirdly, the Chinese government implements a series of rigorous non‐pharmaceutical interventions and measurements which effectively contain the COVID‐19 outbreak, including mask wearing, hand hygiene, aggressive detection, and quarantine and social distance.^10^, ^11^ These above factors might lead to different viruses identified in ARI patients from our study, highlighting the importance the real‐time surveillance of respiratory viruses.

Prevalence in other cities from China appears to be lower than Macao, such as Guangzhou (55.7%),^31^ Shenzhen (14.55%),^32^ Shanghai (24.5%)^33^ and Chengdu (51%).^34^

Fever was a frequent clinical presentation in our virus infection group. Other symptoms associated with the viral respiratory infections included cough, sputum, and nasal obstruction. Although the upper respiratory infections were usually a self‐limited illness with a short duration, the viral infection is sometimes accompanied by a bacterial complication. Additionally, we found that neutrophil and eosinophil might be beneficial to help differentiate those with or without respiratory viral infection. Indeed, consistent with our findings, neutrophils are important innate immune cells that infiltrate lungs rapidly after respiratory insult, while eosinophils are potent secretors for cytotoxic granule proteins and cytokines involved in rapid response to respiratory viral infection.^35^, ^36^

Given the non‐typical clinical presentations, ARI caused by either virus or bacteria were almost indistinguishable. However, molecular diagnosis could provide important information for optimal treatment strategy. Our data showed that empirical administration of antiviral agents or antibiotics might be misleading, suggesting the necessities of rapid diagnosis of respiratory etiology for ARI.

ARI was associated with hospitalization and death, especially in patients with rapidly progressive severe pneumonia.^37^, ^38^ Despite all of our patients recovered eventually, our data also showed that elder people and the patients accompanied by underlying diseases were more vulnerable to pneumonia, presenting with sputum and chest oppression. Our data highlighted special care needs for the aging population and those with pre‐existing diseases.

The presented study has limitations that warrant discussion. Firstly, the clinical specimens were collected during one winter season in 2021 year. Secondly, some bacteria caused ARI such as Streptococcus pneumoniae, non‐typeable Haemophilus influenzae (NTHi), Moraxella catarrhalis, Staphylococcus aureus, Bordetella pertussis, Mycoplasma pneumoniae, and Chlamydia were not detected. Thirdly, we did not able to directly compare the clinical feature and viral etiology responsible for ARI before and after COVID‐19 pandemic.

## 5 CONCLUSIONS

Our study identified the viral etiology and clinical features of ARI patients from fever clinics in winter during COVID‐19, and HRV, PIV‐III, and HCoV‐NL63 were the most prevalent viruses identified in our study. Molecular diagnosis of these ARI patients provides useful information to further understand the epidemiology and clinical feature of respiratory virus during COVID‐19 pandemic.

## CONFLICT OF INTEREST

The authors declare no conflict of interest.

## Supporting information


Table S1.

Table S2.

Table S3.

## Data Availability

The data that support the findings of this study are available from the corresponding author upon reasonable request.
