# Supplementary material for: Clinical features and viral etiology of acute respiratory infection in an outpatient fever clinic during COVID‐19 pandemic in a tertiary hospital in Nanjing, China
Source: J Clin Lab Anal. 2022 Nov 29;36(12):e24778. doi: 10.1002/jcla.24778 (PMC9756996; doi:10.1002/jcla.24778)
Supplement: Supplementary file 1 — Table S1. [file JCLA-36-0-s003.pdf]

Table 1 supplementary. Blood test analysis for ARI patients among pneumonia group versus non-pneumonia group.

	Pneumonia （n=91）	Non-pneumonia (n=151)	P
WBC(10^9/L)	10.89±4.62	9.98±3.72	0.112
Neutrophil (10^9/L)	8.95±4.49	7.99±3.54	0.081
Lymphocyte (10^9/L)	1.21±0.79	1.37±0.91	0.159
Monocyte count (10^9/L)	0.68±0.43	0.67±0.58	0.844
Basophil (10^9/L)	0.015±0.013	0.015±0.013	0.228
Eosinophil (10^9/L)	0.02(0.00,0.08)	0.03(0.01,0.07)	0.261
RBC (10^{12}/L)	4.46±0.69	4.69±0.54	0.004
Hemoglobin(Hb) (g/L)	133.10±23.51	141.30±15.25	0.005
Platelet (10^9/L)	211.30±79.97	224.24±62.20	0.290
