# Supplementary material for: Clinical features and viral etiology of acute respiratory infection in an outpatient fever clinic during COVID‐19 pandemic in a tertiary hospital in Nanjing, China
Source: J Clin Lab Anal. 2022 Nov 29;36(12):e24778. doi: 10.1002/jcla.24778 (PMC9756996; doi:10.1002/jcla.24778)
Supplement: Supplementary file 2 — Table S2. [file JCLA-36-0-s001.pdf]

Table 2 supplementary. Sensitivity analysis of blood test for ARI patients among viral infection group versus non-virus infection group.

	Virus infection (n=52)	Non-Virus infection (n=175)	P
WBC(10^9/L)	9.62±3.40	10.78±4.18	0.071
Neutrophil (10^9/L)	7.49±3.18	8.79±4.07	0.037
Lymphocyte (10^9/L)	1.33±0.66	1.36±0.93	0.854
Monocyte count (10^9/L)	0.66±0.31	0.68±0.56	0.762
Basophil (10^9/L)	0.017±0.013	0.015±0.013	0.223
Eosinophil (10^9/L)	0.06(0.010, 0.150)	0.020(0.010, 0.060)	0.002
RBC (10^{12}/L)	4.63±0.53	4.67±0.56	0.589
Hemoglobin (Hb) (g/L)	139.41±16.95	140.60±16.62	0.657
Platelet (10^9/L)	225.29±72.02	223.12±60.99	0.830
