# Supplementary material for: Clinical features and viral etiology of acute respiratory infection in an outpatient fever clinic during COVID‐19 pandemic in a tertiary hospital in Nanjing, China
Source: J Clin Lab Anal. 2022 Nov 29;36(12):e24778. doi: 10.1002/jcla.24778 (PMC9756996; doi:10.1002/jcla.24778)
Supplement: Supplementary file 3 — Table S3. [file JCLA-36-0-s002.pdf]

Table 3 supplementary. Sensitivity analysis for Blood test of ARI patients among pneumonia group versus non-pneumonia group.

	Pneumonia (n=80)	Non-pneumonia (n=147)	P
WBC(10^9/L)	11.17±4.68	10.16±3.61	0.094
Neutrophil (10^9/L)	9.14±4.57	8.14±3.47	0.090
Lymphocyte (10^9/L)	1.28±0.80	1.39±0.91	0.354
Monocyte count (10^9/L)	0.68±0.34	0.68±0.59	0.962
Basophil (10^9/L)	0.015±0.013	0.016±0.014	0.587
Eosinophil (10^9/L)	0.020(0.000, 0.088)	0.030(0.010, 0.080)	0.254
RBC (10^{12}/L)	4.59±0.58	4.71±0.54	0.142
Hemoglobin(Hb) (g/L)	137.97±18.79	141.62±15.28	0.119
Platelet (10^9/L)	214.78±73.29	228.56±57.01	0.148
